# The prognostic utility of protein C as a biomarker for adult sepsis: a systematic review and meta-analysis

**DOI:** 10.1186/s13054-022-03889-2

**Published:** 2022-01-14

**Authors:** Vanessa Catenacci, Fatima Sheikh, Kush Patel, Alison E. Fox-Robichaud

**Affiliations:** 1 McMaster University, 1280 Main Street, Hamilton, ON L8S 4L8 Canada; 2 University of Toronto, 3359 Mississauga Road, Mississauga, ON L5L 1C6 Canada; 3 Thrombosis and Atherosclerosis Research Institute (TaARI), McMaster University, DBRI C5-106, 237 Barton St East, Hamilton, ON L8L 2X2 Canada

**Keywords:** Sepsis, Biomarker, Protein C, Systematic review, Diagnostic, Prognostic

## Abstract

**Background:**

Sepsis, the dysregulated host response to infection, triggers abnormal pro-coagulant and pro-inflammatory host responses. Limitations in early disease intervention highlight the need for effective diagnostic and prognostic biomarkers. Protein C’s role as an anticoagulant and anti-inflammatory molecule makes it an appealing target for sepsis biomarker studies. This meta-analysis aims to assess the diagnostic and prognostic value of protein C (PC) as a biomarker for adult sepsis.

**Methods:**

We searched MEDLINE, PubMed, EMBASE, CINAHL and Cochrane Library from database inception to September 12, 2021. We included prospective observational studies of (1) adult patients (> 17) with sepsis or suspicion of sepsis that; (2) measured PC levels with 24 h of study admission with; and (3) the goal of examining PC as a diagnostic or prognostic biomarker. Two authors screened articles and conducted risk of bias (RoB) assessment, using the Quality in Prognosis Studies (QUIPS) and the Quality Assessment in Diagnostic Studies-2 (QUADAS-2) tools. If sufficient data were available, meta-analysis was conducted to estimate the standardized mean difference (SMD) between patient populations.

**Results:**

Twelve studies were included, and 8 were synthesized for meta-analysis. Pooled analysis demonstrated moderate certainty of evidence that PC levels were less reduced in sepsis survivors compared to non-survivors (6 studies, 741 patients, SMD = 0.52, 95% CI 0.24–0.81, *p* = 0.0003, *I*^2^ = 55%), and low certainty of evidence that PC levels were less reduced in septic patients without disseminated intravascular coagulation (DIC) compared to those with DIC (3 studies, 644 patients, SMD = 0.97, 95% CI 0.62–1.32, *p* < 0.00001, I^2^ = 67%). PC could not be evaluated as a diagnostic tool due to heterogeneous control populations between studies.

**Conclusion and relevance:**

Our review demonstrates that PC levels were significantly higher in sepsis survivors compared to non-survivors and patients with sepsis but not disseminated intravascular coagulation (DIC). Our evaluation is limited by high RoB in included studies and poor reporting of the sensitivity and specificity of PC as a sepsis biomarker. Future studies are needed to determine the sensitivity and specificity of PC to identify its clinical significance as a biomarker for early sepsis recognition.

*Trial Registration* PROSPERO registration number: CRD42021229786. The study protocol was published in BMJ Open.

**Supplementary Information:**

The online version contains supplementary material available at 10.1186/s13054-022-03889-2.

## Introduction

### Rationale

Sepsis, a life-threatening organ dysfunction caused by a dysregulated host response to infection, is a leading cause of mortality worldwide [1, 2]. With 48.9 million sepsis cases and 11 million sepsis-related deaths in 2017, the disease has a mortality rate of approximately 22% and represents 19.7% of deaths worldwide [2]. To address the high mortality rate, the *Surviving Sepsis* campaign emphasizes the importance of early therapeutic interventions and improved screening for high-risk patients [3].

Diagnosing early-stage sepsis and identifying those at high-risk for mortality remain a challenge due to the disease’s heterogeneous presentation. Sepsis presentation and outcome are affected by multiple factors, including patient characteristics, the causative microorganism and the site of infection [4]. While the current Sepsis-3 definition uses the Sequential Organ Failure Assessment (SOFA) scale to diagnose patients, the use of a highly specific and sensitive diagnostic biomarker could aid timely and appropriate treatment. In addition, the use of SOFA and quick SOFA (qSOFA), as risk stratification models, is limited by low specificity, and the sensitivity and specificity of qSOFA have been shown to vary widely across studies [5–7]. These limitations drive the need for a new rapid and sensitive diagnostic and prognostic test.

Biomarkers are an appealing research target because their rapid quantification has the potential to diagnose disease, predict prognosis and guide early therapeutic interventions. Although the Surviving Sepsis Committee (SSC) highlights the potential value of a biomarker for aiding in the prognosis and diagnosis of sepsis, they do not provide any recommendations for a biomarker when evaluating patients [8]. Identifying an ideal biomarker for sepsis is challenging due to the complex intersection between pro-coagulant, pro-inflammatory and anti-inflammatory mechanisms in sepsis pathology [9, 10]. Given the complex pathophysiology of sepsis, it can be theorized that the optimal biomarker would play a role in multiple septic pathways.

One example of a such a biomarker is protein C (PC), the zymogen of activated protein C (aPC). aPC is a vitamin K-dependent glycoprotein that circulates through the blood plasma [11]. Primarily known as an anticoagulant, aPC negatively regulates the coagulation cascade by preventing fibrin formation, and platelet and coagulation cofactor activation through inhibition of Factor V and VIII [12]. It also contributes to anti-inflammation through upregulation of anti-inflammatory mediators, control of danger-associated molecular patterns and downregulation of leukocyte adhesion and migration [13, 14]. In sepsis, dysregulation of PC contributes to excessive thrombosis and inflammation [11]. Previous research has demonstrated that septic patients display a reduction in endogenous PC levels [15]. This is attributed to increased consumption, decreased protein synthesis in the liver and proteolytic degradation by neutrophil elastase [16].

Given PC’s involvement in sepsis pathology, recent investigations have focused on PC’s utility as a prognostic and diagnostic biomarker in patients with sepsis. To date, the use of PC as a biomarker for sepsis has only been reported in individual clinical studies. Clinicians caring for septic patients require a comprehensive evaluation of PC’s utility as a biomarker for sepsis to inform evidence-based practices.

### Objective

This systematic review and meta-analysis will synthesize existing knowledge and evaluate PC’s utility as a prognostic and diagnostic biomarker in adult sepsis patients.

## Methods

### Protocol/registration

This systematic review and meta-analysis was performed in accordance with the 2020 Preferred Reporting Items for Systematic Reviews and Meta-Analyses (PRISMA) statement [17]. The protocol of this systematic review has been published in BMJ Open [18]. The systematic review was registered in PROSPERO (CRD42021229786).

### Eligibility criteria

We included prospective observational studies that examined one of the following: (1) PC as a diagnostic marker of sepsis or sepsis-induced DIC or (2) PC as a prognostic marker for sepsis-related mortality. Eligible participants were male and female adults (> 17 years of age) with sepsis (including severe sepsis, septic shock or sepsis with DIC) or suspicion of sepsis. Studies enrolling patients with any of the three consensus definitions were included [1, 19, 20]. Only studies measuring PC in the blood of adult sepsis patients within 24 h of study enrollment were included (Table 1).

**Table 1:** PICOS criteria for included studies

Parameter	Inclusion criteria
Population	Adult (> 17 years of age), with sepsis (including severe sepsis, septic shock or sepsis with DIC) or suspicion of sepsis
Intervention	Measurement of PC in the blood within 24 h of study enrollment
Comparator	N/A
Outcomes	*Prognostic outcomes*: (1) PC biomarker levels in survivors vs. non-survivors and (2) the prognostic accuracy of protein C for sepsis-related mortality *Diagnostic outcomes*: (1) PC biomarker levels in septic vs. non-septic patients; (2) the diagnostic accuracy of protein C for sepsis; (3) PC biomarker levels in septic patients with and without DIC; and (4) the diagnostic accuracy of PC for sepsis-induced DIC
Study Design	Prospective observational studies

We excluded retrospective observational studies, abstracts, editorials, poster presentations and non-English studies. Studies on pediatric populations or animal studies were also excluded. We also excluded studies for which there were insufficient data to examine one of the following two: (1) mean PC biomarker levels or (2) the sensitivity, specificity and area under the receiver operating curve (AU-ROC) of PC.

### Information source and search strategy

We initially searched PubMed, EMBASE, CINAHL, Medline and CENTRAL databases from their date of inception to January 20, 2021, and then reran our search September 12, 2021, to include any more recent studies. A full search strategy is detailed in Additional file 1 (See search strategy). The citations of included studies were screened to identify additional studies for inclusion.

### Study selection

We screened studies in a two-step process on Covidence: first by title and abstract, and then by full text, according to the defined inclusion/exclusion criteria. All articles were screened independently in duplicate (VC & KP), and disagreements regarding the eligibility of studies were resolved by discussion or consultation with a third reviewer (FS). We also screened the references of included studies for additional studies.

### Data extraction and management

Two reviewers (VC and KP) extracted study data independently and in duplicate using a standardized data abstraction table, created in Excel. Study authors were contacted for further information if necessary. Any disagreements between reviewers during the extraction were resolved by discussion or consultation with a third reviewer (FS). The following information was extracted from published articles and the corresponding supplemental material: (1) bibliographic details: first author, publication year, study setting, type of study (prognostic or diagnostic) and country; (2) demographic and clinical information: study size, mean age, sepsis definition used, patient population description (severity of sepsis), mortality proportion and follow-up duration for mortality; (3) protein C measurement: time point of measurement and protein C assay used; and (4) study outcomes: Mean biomarker levels, PC threshold values, area under receiver operating curve (AU-ROC), sensitivity, specificity, positive and negative predictive values.

### Assessment of risk of bias

Two reviewers independently performed quality assessments (VC and KP). Agreement between the two reviewers for the assessment of methodological quality was evaluated using Cohen’s kappa statistic. The Quality in Prognostic Studies (QUIPS) tool and the Quality Assessment of Diagnostic Accuracy Studies (QUADAS-2) tool were used for studies evaluating the prognostics and diagnostic outcome, respectively [21, 22]. Any discrepancies were resolved through discussion with a third author (FS). For studies with insufficient information to make a category decision, the RoB was classified as unclear.

### Data analysis

All statistical analyses were performed using RevMan V5.4.1 [23]. We present study and summary estimates as standard mean difference (SMD) for continuous outcomes with 95% confidence intervals. If studies reported mean and standard error, the SD was computed using the formula provided by the Cochrane collaboration [24]. If mean biomarker data were not reported, we used the method by Wan et al*.* to estimate the mean and SD using the median and interquartile range (IQR) or median and range [25]. Heterogeneity was assessed using visual inspection of forest plots, the *I*^2^ statistic and the Chi-squared test for homogeneity (significance at *p* < 0.05). Meta-analyses were performed using fixed effects models if there was no significant heterogeneity (*I*^2^ < 50%); otherwise, a random effects model was used.

The following outcomes were pooled for meta-analysis: (1) SMD in PC levels in sepsis survivors and non-survivors; and (2) SMD in PC levels in sepsis patients with and without DIC. PC differences in septic vs. non-septic patients could not be synthesized for meta-analysis due to heterogeneous control groups. A *p* value of < 0.05 was considered significant.

We planned to conduct H-SROC analysis to evaluate sensitivity and specificity for each outcome [19]; however, a limited number of studies reported the data needed for this analysis. Instead, AUC, sensitivity, specificity, positive predictive value (PPV) and/or negative predictive value (NPV) values reported in the included studies were summarized.

### Subgroup and sensitivity analysis

We performed subgroup analysis on the prognostic outcome to examine 28-day sepsis-related mortality. We planned to conduct subgroup analysis to evaluate only the studies using the recent Sepsis-3 definition; however, insufficient studies were available for this analysis. Many studies began before 2016 and the implementation of the Sepsis-3 definition, and therefore relied on Sepsis-1 and Sepsis-2 definitions for study enrollment.

Sensitivity analysis was performed for our prognostic outcome to assess the effect of removing studies with high RoB. Studies evaluated by QUIPS were determined to be high RoB if they had a “high” distinction for any category or two “moderate” distinctions. Sensitivity analysis was not performed for our diagnostic outcome evaluating the difference in protein C levels between septic patients with and without DIC, as all studies included in meta-analysis had been designated as high RoB.

### Assessment of certainty of the evidence

We assessed the certainty of the evidence for pooled outcomes according to the Grading of Recommendations, Assessment, Development and Evaluation (GRADE) methodology [26]. Assessments were made independently and in duplicate with a third reviewer to resolve conflicts.

## Results

### Study selection

**Fig. 1:**
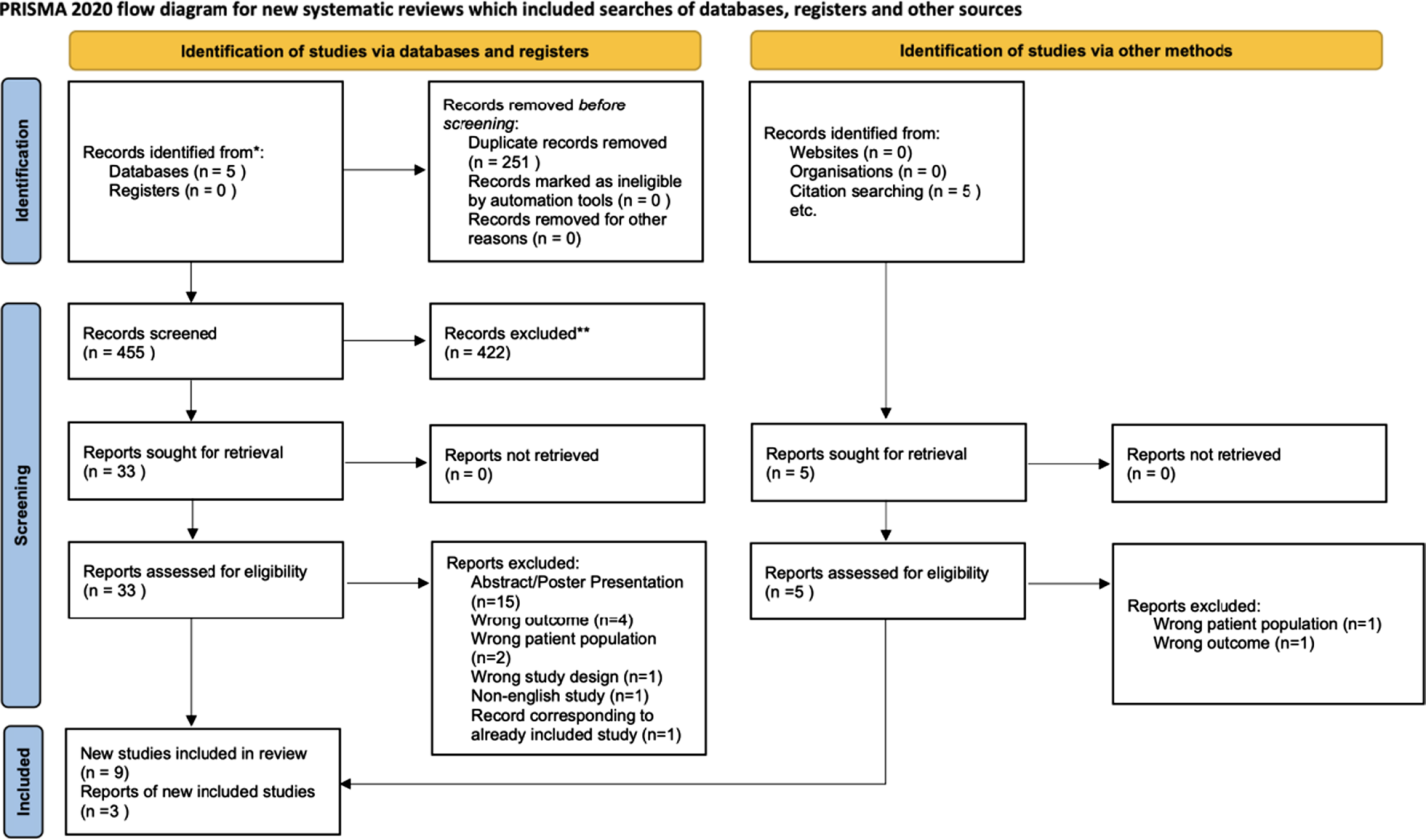
Preferred Reporting Items for Systematic Review and Meta-analyses (PRISMA) 2020 flow chart

The systematic literature search returned 706 articles, with 455 original articles after the removal of duplicates. Figure 1 shows the study selection process. After initial title and abstract and full-text screening, 9 studies were eligible for analysis. In addition, 5 potential articles were identified through citation screening, 3 of which were eligible for inclusion in the study. In total, 12 articles were included in our systematic review [27–38], and 8 articles were combined for meta-analysis [27–35, 38]. The remaining 4 articles were not included in the meta-analysis due to limited available data; however, the study characteristics and available results have been reported in table format [28, 32, 36, 37].

### Study/patient characteristics

Twelve prospective observational studies with a total of 2471 patients were included. Study characteristics are presented in Table 2. All twelve studies were prospective observational and published between 1993 and 2021. Five studies were conducted in North America [28, 31, 33, 35, 37], four in Eastern Asia [27, 29, 32] and three in Europe [30, 34, 38]. The number of participants across studies ranged from 48 to 971 [31, 34].

**Table 2:** Included study characteristics

Study author	Year	Country	Study setting	Prognostic or diagnostic	*N*	Sepsis definition	PC assay	Prognostic outcome	Diagnostic outcome
Chornenki [28]	2020	Canada	MC, ICU	Diagnostic	357	Sepsis-3	ELISA	–	Sepsis + DIC
Masuda [27]	2020	Japan	SC, ED	Diagnostic	107	Sepsis-1	–	–	Sepsis + DIC
Ishikura [29]	2014	Japan	SC, ED	Diagnostic	84	Sepsis-1	Coagulation Analyzer	–	Sepsis
Mihajlovic [30]	2016	Serbia	SC, ED, IDC	Prognostic	150	Sepsis-2	Coagulation Analyzer	28-day mortality	–
Dwivedi [31]	2012	Canada	MC, ICU	Prognostic	80	Sepsis-2	ELISA	Mortality	–
Umemura [32]	2016	Japan	SC, ED	Both	79	Sepsis-1	Coagulation Analyzer	Mortality	Sepsis + DIC
Liaw [33]	2019	Canada	MC, ICU	Prognostic	356	Sepsis-3	ELISA	28-day mortality	–
Lorente [34]	1993	Spain	ICU	Both	48	Sepsis-1	Immunoelectro-phoresis	28-day mortality	Septic shock
Walborn [35]	2020	USA	ICU	Both	103	Sepsis-1	Coagulation Analyzer	28-day mortality	Sepsis + DIC
Koyama [36]	2014	Japan	SC, ICU	Both	77	Sepsis-2	Coagulation Analyzer	28-day mortality	Sepsis + DIC
Shapiro [37]	2009	USA	MC, ED	Both	971	Sepsis-1	ELISA	In-hospital mortality	Severe Sepsis
Karamarkovic [38]	2005	Serbia	SC	Both	59	Sepsis-1	Coagulation Analyzer	Mortality	Surgical sepsis

*MC* multicenter, *SC* single center, *ED* emergency department, *ICU* intensive care unit, *IDC* infectious disease clinic, *DIC* disseminated intravascular coagulation

Protein C levels were measured using two different biochemical techniques. Five studies used antibody-based techniques, such as ELISAs [28, 31, 33, 35, 37], while the remainder of studies measured PC using functional clotting assays [27, 29, 30, 32, 35, 36]. All studies reported their PC measurements as a percentage of healthy PC levels, with the exception of Shapiro et al., which reported PC measurements in ug/mL [37]. Of the studies included, 3 examined our prognostic outcomes [30, 31, 33], 3 examined our diagnostic outcomes [27–29], and the remaining 6 studies examined both outcomes simultaneously [32, 34–38].

The patient characteristics within each study are shown in Table 3. The mean age of the patients varied from 54.9 to 71.7 [27, 37]. SOFA score was reported in 7 studies and ranged from 5.73 to 8.44 [30, 33]. Most studies did not investigate the source of the infection, but those that did identified the lungs, abdomen and urinary tract to be the most common sources of infection [31–33, 36–38]. Finally, the mortality within each study ranged widely, from 7% to 52.1% [34, 37].

**Table 3:** Patient characteristics for included studies. Data presented as mean, mean ± SD or median (Q1,Q3)

Study author	Age	Male (%)	Sepsis severity	SOFA	Infection source (%)	Mortality (%)
Chornenki [28]	63.61 ± 15.24	59.9	Sepsis, sepsis + DIC	8.44 ± 2.64	N.R	23.5
Masuda [27]	71.7 ±	58.8	Sepsis, sepsis + DIC	N.R	N.R	34.57
Ishikura [29]	67.2 ± 17.3	54	Sepsis	7.09	N.R	17.1
Mihajlovic [30]	60.14 ± 16.5	59.3	Sepsis, septic shock	5.73 ± 2.79	N.R	31.3
Dwivedi [31]	63.4 ± 2.24	68.8	Severe sepsis	N.R	Lungs (52.1), blood (23.9), urinary (2.2), abdomen (10.9), skin (4.3), other (2.2), unknown (4.3)	42.5
Lorente [34]	57 ± 7.3	N.R	Severe sepsis, septic shock	N.R	N.R	52.1
Liaw [33]	63.7 ± 14.91	61.2	Sepsis	8.44 ± 2.82	N.R	23.5
Umemura [32]	72 (63–77)	59.5	Sepsis, sepsis + DIC, septic shock	8 (5–10.5)	Lung (14), abdomen (32), urinary tract (29), soft tissue (19), other (6)	16.5
Walborn [35]	57.1 ± 18.6	46.6	Sepsis, sepsis + DIC	5.9 ± 3.7	N.R	14.6
Koyama [36]	69.6 ± 12.9	54.5	Sepsis	9 (7–11)	Pulmonary (19.5), abdominal (55.8), urinary tract (6.5), soft tissue (14.3), blood stream (2.6)	19.5
Shapiro [37]	54.9 ± 19.2	47	Sepsis	N.R	Lower respiratory (31), urogenital (17), soft tissue (12), intra-abdominal (7), catheter-related (5), upper respiratory/suspected viral (14), other (13)	7
Karamarkovic [38]	60.15	N.R	Surgical sepsis	N.R	Abdomen (100)	23

*SOFA* sequential organ failure assessment scale, *N.R* not recorded

### Risk of bias

**Fig. 2:**
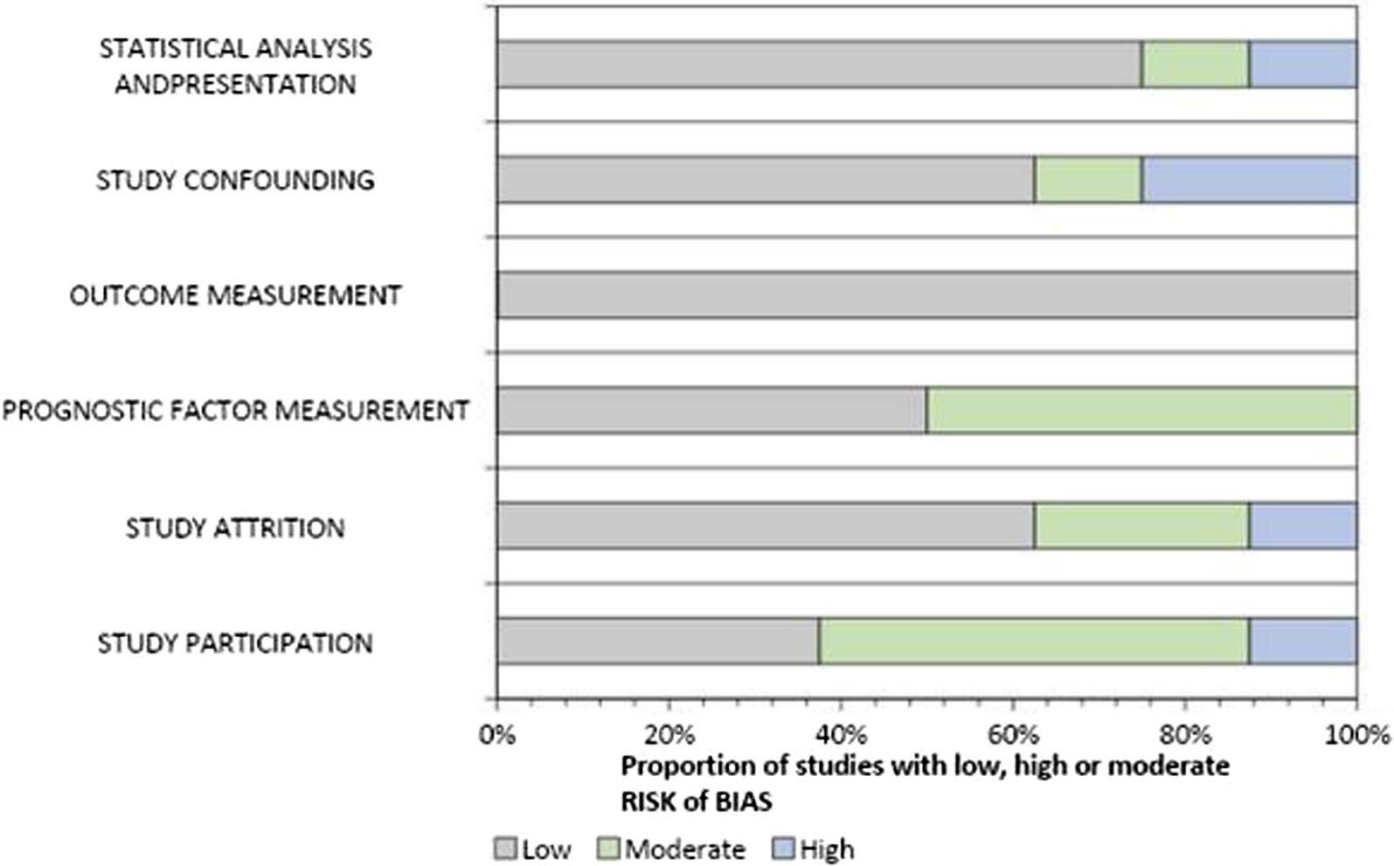
Quality assessment of included prognostic studies, according to the six bias domains of the QUIPS tools

**Fig. 3:**
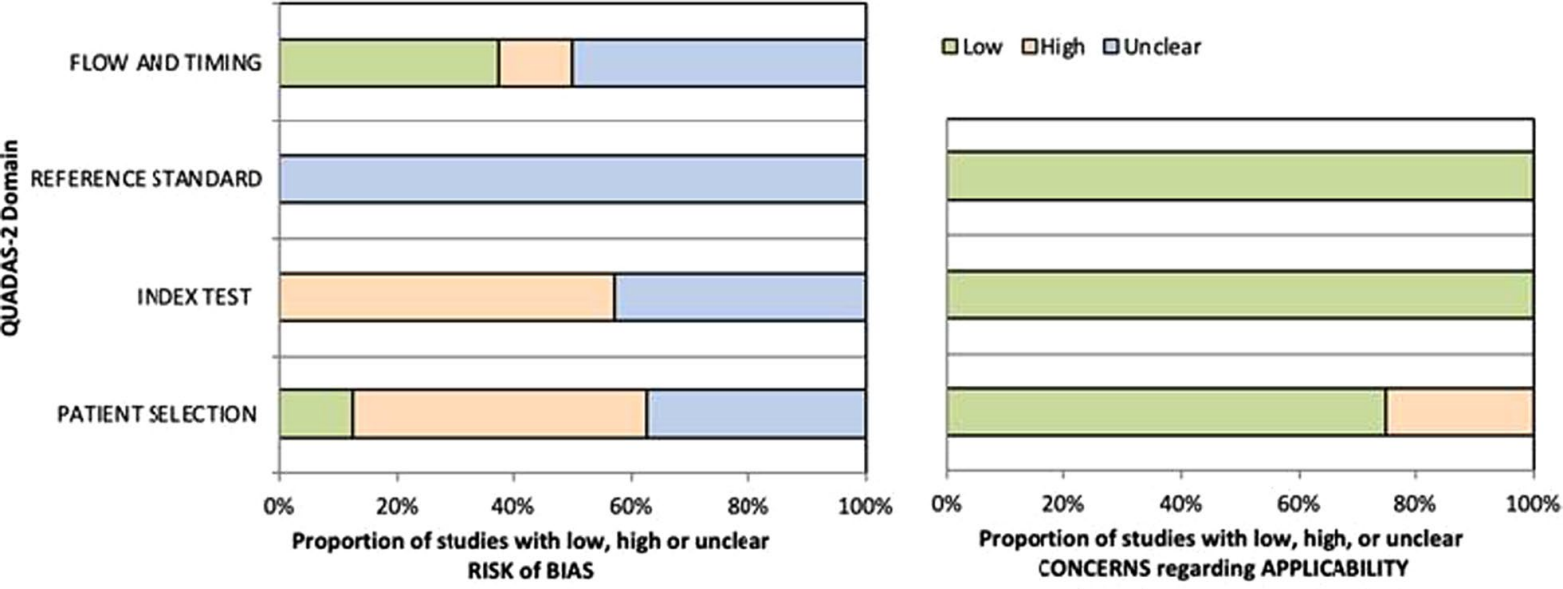
Quality assessment of included diagnostic studies, according to the four domains of the QUADAS-2 tool. **Left:** RoB domains. **Right:** Applicability domains

QUIPS was used to evaluate prognostic studies, while QUADAS-2 was used to evaluate diagnostic studies. The inter-rater agreement for QUIPS and QUADAS-2 was *κ* = 0.74 and *κ* = 0.68, respectively. The overall results of quality assessment are displayed in Figs. 2 and 3.

#### QUIPS

We evaluated 8 prognostic studies using QUIPS. Study participation and prognostic factor (PF) measurement bias was identified as a concern in > 50% of the papers (Fig. 2). Many studies failed to specify whether they followed consecutive or random enrollment, reported no exclusion criteria or were missing information on the time period and location of recruitment. For PF measurement, risk of bias occurred due to missing measurements of PC, data-dependent calculations of sensitivity/specificity and partial reporting of PF information. For study attrition and confounding measurement, > 3 studies had bias concerns. This was due to failure to clarify why some biomarker samples were not taken after enrollment and failure to account for confounding concerns in their study exclusion criteria. Risk of bias for each outcome with justification is found in Additional file 1 (Table S1).

#### QUADAS-2

Eight studies with diagnostic outcomes were evaluated using QUADAS-2. For the patient selection domain, 50% of studies were scored as unclear as they did not provide enough information on their patient enrollment strategy^,^ [27–29], while 3 studies scored high because they used a case–control mechanism when comparing septic patients to healthy control samples [34–36]. Healthy controls are not representative of the patient populations being assessed for sepsis; therefore, the difference in protein C levels between septic and non-septic patients may be overestimated. For the reference standard domain, all studies were marked as unclear as they did not report if the interpretation of the reference standard was blinded to the measurement of the index test. For flow and timing, 5 studies were given a high or unclear risk of bias due to their time intervals between reference and index standards [27, 34, 36–38] (Fig. 3).

All studies were marked as “low” RoB for reference test and index test applicability, as all studies used one of the gold standard sepsis consensus definitions and measured PC within 24 h of hospital admission. Further, 75% of studies scored low in concerns regarding applicability. The remaining two examined specific populations within sepsis (i.e., severe sepsis, septic shock), so there were concerns about the applicability of the studies to our research question. Risk of bias and applicability ratings for each outcome with justification are provided in Additional file 1 (Tables S2 & S3).

### Summary of findings and GRADE certainty

The certainty of evidence is summarized in Additional file 1 (Tables S4 and S5).

### PC biomarker levels in survivors versus non-survivors

**Fig. 4:**
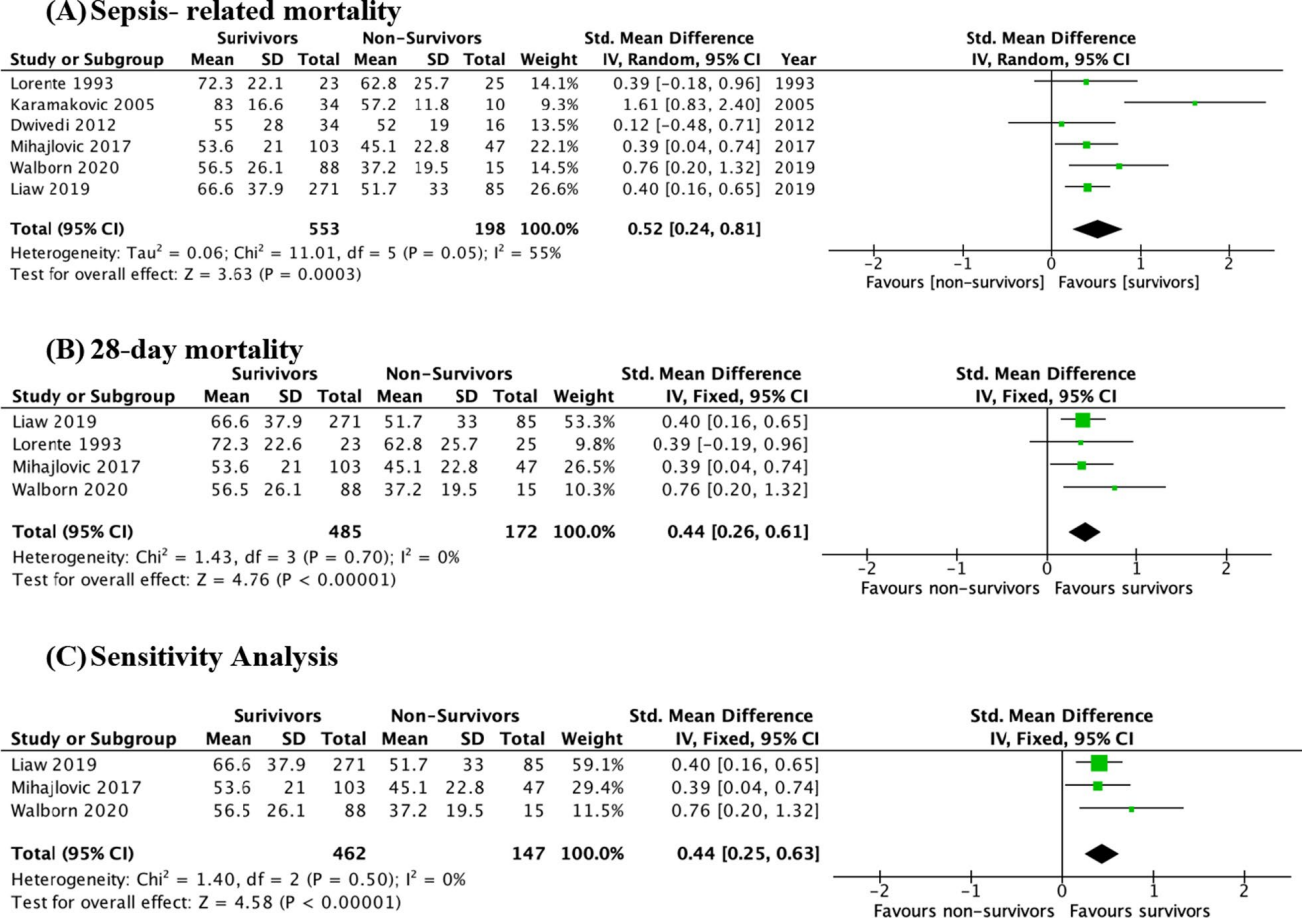
Forest plots of SMD in PC biomarker measurements in survivors vs non-survivors. Standardized mean difference (SMD) estimate favoring survivors indicates that normal PC levels favor survival in sepsis patients. **A** SMD of PC levels in septic survivors vs. non-survivors, **B** SMD of PC in survivors vs. non-survivors for 28-day mortality. **C** Sensitivity analysis conducted by removing high RoB studies

Nine studies examined PC’s ability to predict sepsis-related mortality, with six reporting PC biomarker levels. Of these six studies, three used functional PC assays [30, 35, 37], while three used immunoassays [31, 33, 34]. There was a statistically significant difference between PC levels in survivors and non-survivors (751 patients, SMD 0.52 (95% CI 0.24–0.81), *p* = 0.0003). There was also significant heterogeneity between the studies (*I*^2^ = 55%, *p* = 0.05); therefore, a random effects model was used for analysis (Fig. 4a).

Five studies reported 28-day mortality. There was a statistically significant difference in PC levels between survivors and non-survivors at the 28-day mortality (657 patients, SMD 0.44 (95% CI 0.26–0.61), *p* < 0.00001). Further, the heterogeneity was reduced in this subgroup analysis (*I*^2^ = 0%. *p* = 0.70) (Fig. 4b).

Studies that had a high RoB or two moderate RoB designations using the QUIPS tool were removed for sensitivity analysis. This resulted in reduced heterogeneity (*I*^2^ = 0%. *p* = 0.47) and a statistically significant difference in PC levels between survivors and non-survivors (3 studies, 609 patients, SMD 0.44 (95% CI 0.25–0.63), *p* < 0.00001) (Fig. 4c).

### AUC analysis of protein C as a predictor for mortality

All reported results including sensitivity, specificity, positive predictive value (PPV), negative predictive value (NPV), positive likelihood ratio (LR+) and negative likelihood ratio (LR−) are presented in Table 4.

**Table 4:** ROC analyses for prediction of sepsis-related mortality according to baseline protein C biomarker concentration

Study	Mortality outcome	AUC (95% CI)	Cutoff (%)	Sn	Sp	PPV	NPV	LR+	LR−
Dwivedi [31]	ICU	0.57 (0.43–0.70)	66	–	–	0.66	0.64	–	–
Karamarkovic [38]	Mortality	0.92	66	0.8	0.875	0.67	0.94	6.4	0.23
Koyama [36]	28-day	0.64 (0.45–0.79)	37	0.53	0.75	0.35	0.87	2.12	0.63
Liaw [33]	28-day	–	–	–	–	–	–	–	–
Mihajlovic [30]	28-day	0.65	–	–	–	–	–	–	–
Lorente [34]	28-day	–	–	–	–	–	–	–	–
Umemura [32]	28-day	0. 85 (0.74–0.96)	–	–	–	–	–	–	–
Walborn [35]	28-day	0.71	–	–	–	–	–	–	–

*AUC* area under curve, *Sn* sensitivity, *Sp* specificity, *PPV* positive predictive value, *NPV* negative predictive value, *LR*+ positive likelihood ratio, *LR*− negative likelihood ratio

### Differences in PC biomarker levels in septic versus non-septic patients

Five studies examined the difference between septic patients and their control populations (Additional file 1: Table S6). Three studies used functional PC assays [29, 35, 38] and two used immunoassays [34, 37]. These data could not be synthesized for meta-analysis due to the variability in control groups between the studies. Individually, each study demonstrated a higher PC biomarker level in control patients compared to septic patients, although these values varied widely between studies.

### AUC analysis of protein C as a diagnostic predictor for sepsis

Only Ishikura et al. reported the sensitivity and specificity of PC as a diagnostic tool for sepsis (Sn = 78%, Sp = 81%) [29]. Any other results regarding the sensitivity, specificity, positive predictive value (PPV), negative predictive value (NPV), positive likelihood ratio (LR+) and negative likelihood ratio (LR−) are presented in Additional file 1: Table S7.

### Differences in PC biomarker levels in septic patients with and without DIC

**Fig. 5:**
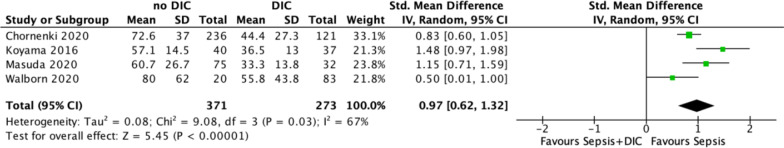
Forest plots of SMD in PC biomarker measurements in septic patients with and without DIC. Standardized mean difference (SMD) estimate favoring septic patients without DIC indicates that normal PC levels favor those without DIC

Four studies examined PC’s diagnostic accuracy for the identification of sepsis-induced DIC. Two studies used a functional assay [32, 35], one used an immunoassay [28], and the last did not state their methodological technique [26]. There was a statistically significant difference between PC levels in septic patients with and without DIC (644 patients, SMD 0.97 (95% CI 0.62–1.32), *p* < 0.00001). There was heterogeneity within the meta-analysis (*I*^2^ = 67%, *p* = 0.03); therefore, a random effects model was used (Fig. 5). Further sensitivity analyses were not conducted, as each study had a high RoB designation in at least one QUADAS-2 category.

### AUC analysis of protein C as a diagnostic predictor for sepsis-induced DIC

Three of the studies conducted ROC analysis, with the AUC values ranging from 0.67 to 0.86 [27, 28]. The results regarding sensitivity, specificity, positive predictive value (PPV), negative predictive value (NPV), positive likelihood ratio (LR+) and negative likelihood ratio (LR−) are presented in Table 5.

**Table 5:** ROC analyses for diagnosis of sepsis + DIC according to baseline PC biomarker concentration

Study	Diagnostic outcome	AUC (95% CI)	Cutoff (%)	Sn	Sp	PPV	NPV	LR+	LR−
Walborn [35]	Sepsis + DIC	–	–	–	–	–	–	–	–
Masuda [27]	Sepsis + DIC	0.86 (0.82–0.90)	42	0.8	0.77	0.6	0.91	3.51	0.26
Koyama [36]	Sepsis + DIC	0.85 (0.76–0.91)	46	0.81	0.79	0.79	0.82	3.86	0.24
Chornenki^28^	Sepsis + Pre-DIC	0.67 (0.58–0.73)	51	–	–	–	–	–	–

*AUC* area under curve, *Sn* sensitivity, *Sp* specificity, *PPV* positive predictive value, *NPV* negative predictive value, *LR*+ positive likelihood ratio, *LR*− negative likelihood ratio

## Discussion

Sepsis remains a global health concern and the lack of accurate diagnostic and prognostic criteria for sepsis remains a huge limitation in early sepsis treatment and risk management [39]. Although the *Surviving Sepsis Campaign* acknowledges the value of biomarkers for septic patient treatment, the 2021 updated guidelines provide no recommendation for the use of any biomarker in the prognosis or diagnosis of sepsis [3]. This systematic review and meta-analysis is the first comprehensive meta-analysis to date that assesses the diagnostic and prognostic accuracy of PC as a biomarker for sepsis. Using rigorous search criteria, this study identified only prospective observational studies to minimize RoB from study design. Further, studies were extensively evaluated using QUIPS and QUADAS-2, which were specifically developed to assess the RoB in prognostic and diagnostic studies, respectively. While we were unable to synthesize data examining PC was a diagnostic marker for sepsis due to study heterogeneity, we identified a large effect size with low certainty demonstrating PC levels are less reduced in septic patients without DIC compared to those with DIC. Further, the meta-analysis demonstrated a moderate effect size with moderate certainty for differences in PC levels within 24 h of sepsis diagnosis in survivors compared to non-survivors. Sensitivity and subgroup analyses failed to demonstrate that the quality of studies or primary outcome affected the prognostic value of PC. Overall, these results indicate that the sensitivity and specificity of protein C should be further investigated as a measure to guide clinical evaluations in identifying sepsis-related DIC and sepsis survival outcomes.

Dysregulation of protein C contributes to excessive thrombosis and inflammation seen in sepsis patients. The cytokine storm produced in early innate immune response decreases the production of negative acute-phase proteins, such as PC [40, 41]. PC levels decrease further due to proteolytic degradation by neutrophil elastase [16]. As sepsis progresses, protein C levels remain low due to impaired transcription, and aPC levels begin to decrease. The release of histones from neutrophil extracellular traps (NETs) impairs thrombin–thrombomodulin binding interaction, a requirement for PC activation [42]. Further, pro-inflammatory cytokines cause increases in soluble endothelial protein C receptor (sEPCR), which binds to PC and acts as a competitive inhibitor to its activation [43]. This reduction in PC and aPC over the course of sepsis causes excessive coagulation and inflammation. Given PC’s clear involvement in sepsis pathology, and limited current knowledge as a diagnostic biomarker, it warrants future in-depth investigation.

There are several limitations to our meta-analysis. We were unable to conduct H-SROC analysis as originally planned due to limited reporting on the AUC, sensitivity and specificity of PC as a prognostic or diagnostic biomarker. Further, we were unable to evaluate heterogeneity by conducting subgroup analysis based on sepsis definition, geographical location or decade of study publication due to a limited number of studies. The inability to analyze studies that use only the most recent Sepsis-3 definition highlights the need for future studies evaluating PC’s effectiveness as a biomarker. In addition, our study quality assessment demonstrated high RoB in many of the studies included. This was largely due to underreporting of information on patient selection, lack of index text blinding, reporting of AUC values that were based on data-dependent cutoffs and missing patient biomarker samples. Finally, this study identified that papers generally use one of two ways to measure protein C: immunoassays to evaluate PC antigen levels and coagulation assays to measure PC functional activity levels. We chose to combine all studies for meta-analysis regardless of the assay used to measure PC, as they all presented their PC measurements as a % of healthy controls. However, this should be regarded with caution, and future studies should make a clearer distinction the method of protein C measurement used.

Despite these limitations, the results demonstrated that protein C measurements offer guidance as an indicator of the presence of sepsis-induced DIC and risk of mortality. Further, this meta-analysis helps to inform future researchers of the potential of PC as a part of a multi-biomarker panel for sepsis prognosis. Going forward, studies are needed to evaluate whether PC can be used as a biomarker in early diagnostic settings, such as the emergency department [44]. Further, future studies must include more robust data on the sensitivity, specificity, PPV and NPV of PC as a biomarker to ascertain the clinical utility of PC for sepsis prognosis and/or diagnosis.

## Conclusion

Our systematic review and meta-analysis of current literature suggests that PC levels are less reduced in sepsis survivors compared to non-survivors and in septic patients without DIC compared to those with DIC. There are insufficient data in the current literature to determine whether PC levels are different in septic vs. non-septic patients. More robust studies on the sensitivity and specificity of PC as a biomarker are needed to determine clinical significance of this biomarker as a prognostic or diagnostic tool for sepsis.

## Supplementary Information

**Additional file 1**. Search strategy and Table S1–S7. The prognostic utility of protein C as a biomarker for adult sepsis: a systematic review and meta-analysis - Additional data.

## Data Availability

All data generated and/or analyzed during the current study are included within the published article and its additional files.

## Abbreviations

AU-ROC: Area under the receiver operating characteristic
CI: Confidence interval
CINAHL: Cumulative Index to Nursing and Allied Health Literature
DIC: Disseminated intravascular coagulation
ED: Emergency department
ELISA: Enzyme-linked immunoassay
GRADE: Grading of Recommendations, Assessment, Development and Evaluation
HSROC: Hierarchical summary receiver operating characteristic
ICU: Intensive care unit
IDC: Infectious disease center
LR+: Positive likelihood ratio
LR−: Negative likelihood ratio
MC: Multicenter
NPV: Negative predictive value
PC: Protein C
PRISMA: Preferred Reporting Items for Systematic Reviews and Meta-Analyses
PPV: Positive predictive value
RoB: Risk of bias
SC: Single center
SD: Standard deviation
SMD: Standardized mean difference
Sn: Sensitivity
SOFA: Sequential organ failure assessment scale
Sp: Specificity
Q1: Quartile 1
Q3: Quartile 3
