# Supplementary material for: The prognostic utility of protein C as a biomarker for adult sepsis: a systematic review and meta-analysis
Source: Crit Care. 2022 Jan 14;26:21. doi: 10.1186/s13054-022-03889-2 (PMC8760778; doi:10.1186/s13054-022-03889-2)
Supplement: Supplementary file 1 — Additional file 1. Search strategy and Table S1–S7. The prognostic utility of protein C as a biomarker for adult sepsis: a systematic review and meta-analysis - Additional data. [file 13054_2022_3889_MOESM1_ESM.pdf]

Additional File 1: The Prognostic Utility of Protein C as a Biomarker for Adult Sepsis: A systematic review and meta-analysis- additional data

Authors:

1. Vanessa Catenacci BSc, (MD candidate)
   Email: vanessa.catenacci@medportal.ca
   Institutional Address: McMaster University, 1280 Main Street, Hamilton, ON, L8S 4L8
2. Fatima Sheikh BSc, (MSc candidate)
   Email: sheikf9@mcmaster.ca
   Institutional Address: McMaster University, 1280 Main Street, Hamilton, ON, L8S 4L8
3. Kush Patel BHSc, (MMI candidate)
   Email: kusha.patel@mail.utoronto.ca
   Institutional Address: University of Toronto, 3359 Mississauga Road, Mississauga, ON, L5L 1C6
4. Alison E. Fox-Robichaud MSc, MD, FRCPC
   Email: afoxrob@mcmaster.ca
   Institutional Address: McMaster University, 1280 Main Street, Hamilton, ON, L8S 4L8

All authors are affiliated with McMaster University.

Corresponding author:
Dr. Alison-Fox Robichaud
DBRI C5-106
237 Barton St. East
Thrombosis & Atherosclerosis Research Institute (TaARI)
Hamilton, ON L8L 2X2
Telephone: (905)-521-2100 ext 40742

Email: afoxrob@mcmaster.ca

This supplementary appendix provides:

1. Search Strategy
2. Quality assessment of the included studies
3. GRADE evaluation
4. Evaluation of Protein C as a diagnostic tool for adult sepsis

1. **Search Strategy**

Search strategies for the different databases ran on January 20th, 2021.

MEDLINE

#1 (Sepsis):ti,ab,kw OR (Septic):ti,ab,kw OR (Severe sepsis):ti,ab,kw OR (Septic shock):ti,ab,kw AND (Septicemia):ti,ab,kw (Word variations have been searched)
#2 (Protein C):ti,ab,kw (Word variations have been searched)
#3 (Biomarker):ti,ab,kw (Word variations have been searched)
#4 #1 AND #2 AND #3

EMBASE

#1 (Sepsis):ti,ab,kw OR (Septic):ti,ab,kw OR (Severe sepsis):ti,ab,kw OR (Septic shock):ti,ab,kw AND (Septicemia):ti,ab,kw
#2 (Protein C):ti,ab,kw
#3 (Biomarker):ti,ab,kw
#4 #1 AND #2 AND #3

PubMed

((((((sepsis[Title/Abstract]) OR (septic[Title/Abstract])) OR (septic shock[Title/Abstract])) OR (septicemia[Title/Abstract])) OR (severe sepsis[Title/Abstract])) AND (biomarker[Title/Abstract])) AND (Protein C[Title/Abstract])

CINAHL

#1 (Sepsis):ti,ab,kw OR (Septic):ti,ab,kw OR (Severe sepsis):ti,ab,kw OR (Septic shock):ti,ab,kw AND (Septicemia):ti,ab,kw (Word variations have been searched)
#2 (Protein C):ti,ab,kw (Word variations have been searched)
#3 (Biomarker):ti,ab,kw (Word variations have been searched)

#4 #1 AND #2 AND #3

Cochrane Library

#1 (Sepsis):ti,ab,kw OR (Septic):ti,ab,kw OR (Severe sepsis):ti,ab,kw OR (Septic shock):ti,ab,kw AND (Septicemia):ti,ab,kw (Word variations have been searched)

#2 (Protein C):ti,ab,kw (Word variations have been searched)

#3 (Biomarker):ti,ab,kw (Word variations have been searched)

#4 #1 AND #2 AND #3

2. Quality assessment of included studies

Supplementary Table 1: QUIPS Risk of bias with justification for studies evaluating PC as a prognostic indicator of sepsis-related mortality

Author	Outcome	Study participation	Study Attrition	Prognostic Factor Measurement	Outcome Measurement	Study Confounding Measurement	Statistical Analysis and Presentation
Liaw	28-day mortality	Low[a]	Low[d]	Low[g]	Low[i]	Low[j]	Low[m]
Walborn	28-day mortality	Moderate[b]	Low[d]	Low[g]	Low[i]	Low[j]	Low[m]
Mihajlovic	28-day mortality	Moderate	Low[d]	Low[g]	Low[i]	Low[j]	Low[m]
Koyama	28-day mortality	Low[a]	Low[d]	Low[g]	Low[i]	Low[j]	Low[m]
Umemura	28-day mortality	Low[a]	Low[d]	Moderate[h]	Low[i]	High[k]	Moderate[n]
Dwivedi	ICU mortality	Moderate[b]	High[e]	Moderate[h]	Low[i]	Low[j]	Moderate[n]
Karamakovic	In-hospital mortality	Moderate[b]	Moderate[f]	Moderate[h]	Low[i]	Moderate[l]	Low[m]
Lorente	Mortality	High[c]	Moderate[f]	Moderate[h]	Low[i]	High[k]	Low[m]

[a]Recruitment period/time frame specified, detailed inclusion/exclusion criteria and description of baseline characteristics suggest no problem with study participation

[b]Studies had at least 2/3 of the following concerns: (1) no mention of recruitment period (2) partial or no mention of place of recruitment (3) Partial or no information on the method used to identify the study population, with no specification on if patients were randomly or consecutively enrolled

[c]This study had no exclusion criteria stated for study enrollment. Information on recruitment period and place of recruitment was also missing.

[d]Outcome data was available for all participants

[e] Only 62.5% of enrolled participants had samples available for analysis. Differences between patients who had samples available and those who did not was not examined.

[f]Both studies did not specify whether all baseline participants were followed up for sample collection/analysis after study enrollment.

[g]Clear definition of Protein C measurement and assay used, >90% of all samples were available for analysis, continuous variables (ie: biomarker levels) and/or non-data dependent cut-offs were reported

[h]Studies had at least one of the following issues: (1) The only measurement for Protein C provided in the study was an AUC value calculated from a data-dependent cut-off (2) >20% of prognostic factor measurements were not available for analysis (3) Study did not specify proportion of PF information available for analysis

[i]Clear outcome definition relating to mortality

[j]Detailed study inclusion/exclusion criteria accounted for any confounding concerns. Any other confounders related to study design or population was highlighted in their discussion.

[k]Both studies had 2 concerns: (1) Limited/inexistent exclusion criteria did not account for potential confounding factors (2) Both studies examined only severe sepsis and/or septic shock patients, and did not account for the potential generalizability limitations in their analysis

[l]The paper examines only surgical abdominal sepsis patients. There is no reference in their results or discussion to the potential confounding impact this might have to the generalizability of their results.

[m]Studies presented their analytical strategy sufficiently in the methods section. All intended statistical analyses were carried out and reported in the results section.

[n]Both studies reported insufficient information for analysis of PC. Dwivedi specified that PC levels in survivors and non-survivors were statistically different, but did not provide the corresponding biomarker levels. Umemura provided an AUC value for PC as a prognostic biomarker, but did not specify any additional information.

Supplementary Table 2: QUADAS-2 Risk of bias with justification for studies evaluating PC as diagnostic indicator for sepsis and sepsis-induced DIC

Author	Outcome	Patient Selection	Index Test	Reference Standard	Flow/Timing
Koyama	Sepsis-induced DIC	Low[a]	High[e]	Unclear[g]	Unclear[h]
Masuda	Sepsis-induced DIC	Unclear[b]	High[e]	Unclear[g]	Unclear[h]
Chornenki	Sepsis induced pre-DIC	Unclear[b]	High[e]	Unclear[g]	Low[i]
Ishikura	Sepsis	Unclear[b]	High[e]	Unclear[g]	Low[i]
Lorente	Septic Shock	High[c]	Unclear[f]	Unclear[g]	High[j]
Karamakovic	Intra-abdominal sepsis	High[d]	High[e]	Unclear[g]	Unclear[h]
Walborn	Sepsis, Sepsis-induced DIC	High[c]	Unclear[f]	Unclear[g]	Unclear[h]
Shapiro	Severe Sepsis	Unclear	Unclear[f]	Unclear[g]	Low[i]

[a]Study provided details on patient enrollment, avoided a case-control mechanism, avoided inappropriate exclusions of patients eligible

[b]Study did not provide details on whether patient enrollment was consecutive/random/etc., therefore an accurate bias determination could not be made

[c]when evaluating PC as a diagnostic indicator, they compared septic patients matched to healthy controls

[d]The study excluded those with APACHE scores of >15, thereby excluding patients with increased severity of sepsis and affecting the rate of mortality

[e]There was no pre-specific Protein C cut-off value when evaluating PC sensitivity/specificity. The cut-off reported in the papers was the one that provided the highest AUC value.

[f]These studies reported mean biomarker levels rather than AUC data, so cut-offs were not calculated. However, it is unclear if the index test was interpreted while blinded to the patient outcome.

[g]Studies did not report if the interpretation of the reference standard was blinded to the measurement of the index test

[h]Studies stated that biomarker sample was taken within 24 hours of patient diagnosis, but this specific time interval may vary for each patient and affect PC levels

[i]All studies took biomarker sample upon study enrollment, all patients received the same reference standard, and all patients were included in the analysis

[j]Study took PC biomarker samples at 8am the following day regardless at what time the patient was diagnosed with septic shock

Supplementary Table 3: QUADAS-2 Applicability with justification for studies evaluating PC evaluating PC as diagnostic indicator for sepsis and sepsis-induced DIC

Author	Outcome	Patient Selection	Index Test	Reference Standard
Koyama	Sepsis-induced DIC	Low[a]	Low[d]	Low[d]
Masuda	Sepsis-induced DIC	Low[a]	Low[d]	Low[d]
Chornenki	Sepsis induced pre-DIC	Low[a]	Low[d]	Low[d]
Ishikura	Sepsis	Low[a]	Low[d]	Low[d]
Lorente	Septic Shock	High[b]	Low[d]	Low[d]
Karamakovic	Intra-abdominal sepsis	High[c]	Low[d]	Low[d]
Walborn	Sepsis, Sepsis-induced DIC	Low[a]	Low[d]	Low[d]
Shapiro	Severe Sepsis	Low[a]	Low[d]	Low[d]

[a]No major applicability concerns

[b]Exposure patients enrolled in this study were those with septic shock, which may bias the data as it only includes more severe cases of sespsis

[c]Exposure patients enrolled in this study were those with abdominal sepsis, which may bias the data as it does not include data for other sources of sepsis

[d]No major applicability concerns

3. **GRADE Evaluation**

Supplementary Table 4. Grading of Recommendations Assessments, Developments and Evaluations (GRADE) approach for assessing certainty of evidence of study outcomes for sepsis-related mortality

Certainty assessment							№ of patients		Effect	Certainty	Importance
**№ of studies**	**Study design**	**Risk of bias**	**Inconsistency**	**Indirectness**	**Imprecision**	**Other considerations**	**Survivors**	**Non-survivors**	**Relative (95% CI)**		
**PC Biomarker Measurement for Sepsis-Related Mortality**											
6	observational studies	not serious[a]	not serious	not serious	serious [b]	none	542/751 (72.2%)	209/751 (27.8%)	**SMD 0.52 (0.24-0.81)**	⊕⊕⊕◯ Moderate	CRITICAL

[a]Sensitivity analysis excluding high RoB studies had no effect on the conclusion of the summary estimate

[b]All studies evaluated in this meta-analysis were considered to be high RoB

Supplementary Table 5. Grading of Recommendations Assessments, Developments and Evaluations (GRADE) approach for assessing certainty of evidence of study outcomes for sepsis-induced DIC

Certainty assessment							№ of patients		Effect	Certainty	Importance
**№ of studies**	**Study design**	**Risk of bias**	**Inconsistency**	**Indirectness**	**Imprecision**	**Other considerations**	**DIC**	**No DIC**	**Relative (95% CI)**		
**PC Biomarker Measurement for Sepsis-induced DIC**											
4	observational studies	Very serious [c]	not serious	not serious	not serious	none	273/644 (42.4%)	371/644 (57.6%)	**SMD 0.97 (0.62-1.32)**	⊕⊕◯◯ LOW	IMPORTANT

[c]Two studies only examined Protein C levels in a subset of septic patients based on sepsis severity and origin of infection

4. **Evaluation of Protein C as a Diagnostic Tool for Sepsis**

Supplemental Table 6: Baseline Protein C biomarker levels in septic vs. control patients.

Study	Exposure	Control	PC Mean Exposure	SD	(N)	PC Mean Control	SD	(N)
**Ishikura**[26]	Sepsis	Non-septic patients	36.4	24.5	43	75.9	32.7	39
**Lorente**[31]	Septic shock	Healthy volunteers	67.3	24.51	48	96.3	24.51	30
**Karamarkovic**[35]	Surgical Sepsis	Non-septic hernia repair patients	77.1	15.6	44	92.5	6.5	15
**Shapiro**[34]	Severe Sepsis	Non-septic patients	2.26*	1.02*	506	2.94*	1.04*	465*
**Walborn**[32]	Sepsis	Healthy volunteers	60.5	47.5	103	98	18	50

PC biomarker levels presented as % of healthy control unless otherwise stated. (*Protein C measurements presented as ug/mL)

Supplemental Table 7: ROC analyses for diagnosis of sepsis according to baseline) PC biomarker concentration

Study	Diagnostic Outcome	AUC (95% CI)	Cut-off (%)	Sn	Sp	PPV	NPV	LR+	LR-
**Ishikura**[26]	Sepsis	0.834	47	0.78	0.81	0.83	0.76	4.1	0.3
**Karamarkovic**[35]	Surgical Sepsis	-	-	-	-	-	-	-	-
**Lorente**[31]	Septic shock	-	-	-	-	-	-	-	-
**Shapiro**[34]	Severe Sepsis	0.69 (0.66-0.72)	-	-	-	-	-	-	-
**Walborn**[32]	Sepsis	-	-	-	-	-	-	-	-

AUC Area under curve, *Sn* Sensitivity, *Sp* Specificity, *PPV* Positive Predictive Value, *NPV* Negative Predictive Value, *LR+* Positive Likelihood Ratio, *LR-* Negative Likelihood Ratio
